# Simple ab externo lens capsule removal in intraocular lens reuse and intrascleral fixation for intracapsular lens dislocation: A case report

**DOI:** 10.1097/MD.0000000000040840

**Published:** 2024-12-06

**Authors:** Suguru Nakagawa, Kiyoshi Ishii

**Affiliations:** a Department of Ophthalmology, Saitama Red Cross Hospital, Saitama, Japan; b Department of Ophthalmology, Saitama Medical Center, Jichi Medical University, Saitama, Japan.

**Keywords:** intracapsular lens dislocation, intraocular lens reuse, intrascleral fixation, lens capsule removal

## Abstract

**Background:**

This study aimed to report and recall a simple method to remove the lens capsule ab externo when performing intrascleral fixation of an intracapsular intraocular lens (IOL) dislocation with reuse of the IOL.

**Case presentation:**

A 43-year-old Japanese male patient underwent pars plana vitrectomy, phacoemulsification, and IOL fixation for rhegmatogenous retinal detachment in the right eye 10 years prior. A 3-piece IOL was intraocularly fixed during the initial procedure. In December 2023, the patient presented with intracapsular IOL dislocation in his right eye, causing the IOL to descend into the vitreous cavity enveloped by its entire capsule. During surgery, an intravitreal dislocated IOL was placed over the iris with the entire capsule. The haptics on 1 side of the IOL were placed outside the eye through the corneoscleral wound. Subsequently, the lens capsule and Soemmerring’s ring surrounding the haptics were removed. After the haptics were placed back over the iris, the same extraction procedure was performed for the remaining haptics. The IOL was intrasclerally fixed using a flange technique through the intrascleral tunnel at the 4 and 10 o’clock positions. One week postsurgery, the best-corrected visual acuity of the right eye was 20/16, and the corneal endothelial cell density was recorded as 2923 cells/mm^2^ (preoperative: 1650 cells/mm^2^).

**Discussion and conclusions:**

In cases of 3-piece IOL dislocation, employing the ab externo technique for lens capsule extraction has been proven to be a straightforward and efficient method. This approach facilitates the removal of the lens capsule around the IOL. If subsequent damage to the IOL is identified, it allows for easy conversion to replacement.

## 1. Introduction

In treating intraocular lens (IOL) dislocation,^[1]^ 2 primary methods are typically employed: 1 involves the removal of the dislocated IOL followed by replacement with a new IOL, while the other entails the reutilization of the dislocated IOL.^[2,3]^ The fixation of the IOL to the sclera is achieved through the use of nonabsorbable sutures.^[3,4]^

Kim et al^[5]^ demonstrated the efficacy of intrascleral fixation for the reutilization of dislocated IOL. Their study primarily addressed cases involving extracapsular dislocation of the IOL. Furthermore, cases of intracapsular IOL dislocation with reuse of the IOL and intrascleral fixation were reported by Baba et al.^[6]^ However, when intracapsular IOL dislocation is managed with intrascleral fixation, the extraction of the lens capsule surrounding the IOL becomes imperative for the reuse of the IOL.^[6]^ With the widespread use of sutureless intrascleral IOL fixation using the 25-gauge (G) vitrectomy system, especially in Japan, where surgery is often performed by a vitrectomy surgeon, the lens capsule around the IOL that has fallen into the vitreous cavity is commonly removed using a vitrectomy cutter inside the vitreous cavity. In such cases, it is usually necessary to remove the lens capsule around the IOL using forceps and a vitreous cutter under chandelier illumination, ensuring no damage to the IOL. In certain cases, the removal of the lens capsule may necessitate manipulations that are intricate and time-consuming, such as adjusting the cut rate of the vitreous cutter or extracting Soemmering’s ring through the sclerocorneal wound. Furthermore, even after such painstaking removal of the lens capsule, the IOL may be damaged and eventually need to be replaced.

This study aimed to present and recall a simple method to remove the lens capsule ab externo when intrascleral fixation of an intracapsular IOL dislocation was performed with the intention of reusing the IOL.

## 2. Case presentation

A 43-year-old Japanese male patient underwent pars plana vitrectomy (PPV), phacoemulsification, and IOL fixation for rhegmatogenous retinal detachment in the right eye a decade earlier in 2013. A 3-piece IOL (NX-70, Santen, Osaka, Japan), with an optic diameter of 7 mm, was intracapsularly implanted. The patient experienced difficulty in vision in his right eye after being struck by a soccer ball. Subsequently, he visited our department in December 2023 owing to the dislocation of the intracapsular IOL in his right eye. The IOL descended into the vitreous cavity and was enclosed by its entire capsule. The best-corrected visual acuity and intraocular pressure in the right and left eyes were 20/16 and 20/16, and 15 and 14 mm Hg, respectively. The axial lengths were 28.32 and 27.16 mm in the right and left eyes, respectively.

**Figure 1.**
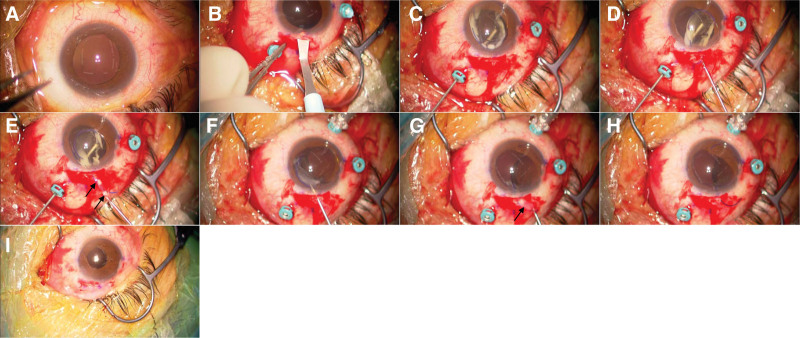
Intraoperative findings. (A) Photograph taken at the beginning of the surgery. The intraocular lens (IOL) dropped into the vitreous cavity and was not visible.(B) A 25-gauge vitreous port was created, and a 2.4-mm corneoscleral incision was made from the upper nasal side. (C) The intravitreally intracapsular dislocated IOL was placed over the iris with the entire capsule. (D) The IOL haptics were placed outside the eye through the corneoscleral wound. (E) The lens capsule, proliferated lens cortex, and Soemmering’s ring were easily removed. The arrows indicate the lens capsule. (F) The IOL was rotated 180°, and the other lens haptics were placed outside the eye through the corneoscleral wound. (G) The lens capsule, proliferated lens cortex, and Soemmering’s ring were easily removed. The arrow indicates the lens capsule. (H) The IOL was exposed after the lens capsule, proliferated lens cortex, and Soemmering’s ring were removed. (I) Photograph of the anterior segment at the end of the surgery. No iris damage was observed. IOL = intraocular lens.

At the beginning of the surgery (Fig. 1A, Video S1, Supplemental Digital Content, http://links.lww.com/MD/O128), under sub-Tenon anesthesia with 0.75% anapain, a 25-G vitreous port was created, and a corneoscleral incision was made from the upper nasal side (Fig. 1B, Video S1, Supplemental Digital Content, http://links.lww.com/MD/O128), followed by the injection of viscoelastic material (SHELLGAN, Santen, Osaka, Japan) to form an anterior chamber. Subsequently, an intravitreally dislocated IOL was placed over the iris with the entire capsule using a vitreous cutter and lens hook (Fig. 1C, Video S1, Supplemental Digital Content, http://links.lww.com/MD/O128). The haptics on 1 side of the IOL were placed outside the eye through the corneoscleral wound (Fig. 1D, Video S1, Supplemental Digital Content, http://links.lww.com/MD/O128). Subsequent steps included the easy removal of the lens capsule and Soemmering’s ring around the haptics (Fig. 1E, Video S1, Supplemental Digital Content, http://links.lww.com/MD/O128). The haptics were then placed back over the iris and the IOL was rotated by 180° (Fig. 1F, Video S1, Supplemental Digital Content, http://links.lww.com/MD/O128). The remaining haptics of the IOL were placed outside the eye through the corneoscleral wound (Fig. 1F–H, Video S1, Supplemental Digital Content, http://links.lww.com/MD/O128). Subsequent steps included the easy removal of the lens capsule and Soemmering’s ring around the haptics (Fig. 1G, H, Video S1, Supplemental Digital Content, http://links.lww.com/MD/O128). Intrascleral fixation of the IOL was achieved using a flange technique and needle method, which used a 30G thin-walled needle^[7,8]^ to reposition the haptics 2 mm from the corneal limbus at the 4 and 10 o’clock positions. A 3-port PPV was performed using a 25-G vitrectomy cutter. After the core vitrectomy, a few partially dropped lens epithelial proliferations were processed using a cutter. Surgery was completed after the periphery was checked with pressure and the absence of retinal tears or detachment was confirmed (Fig. 1I, Video S1, Supplemental Digital Content, http://links.lww.com/MD/O128).

**Figure 2.**
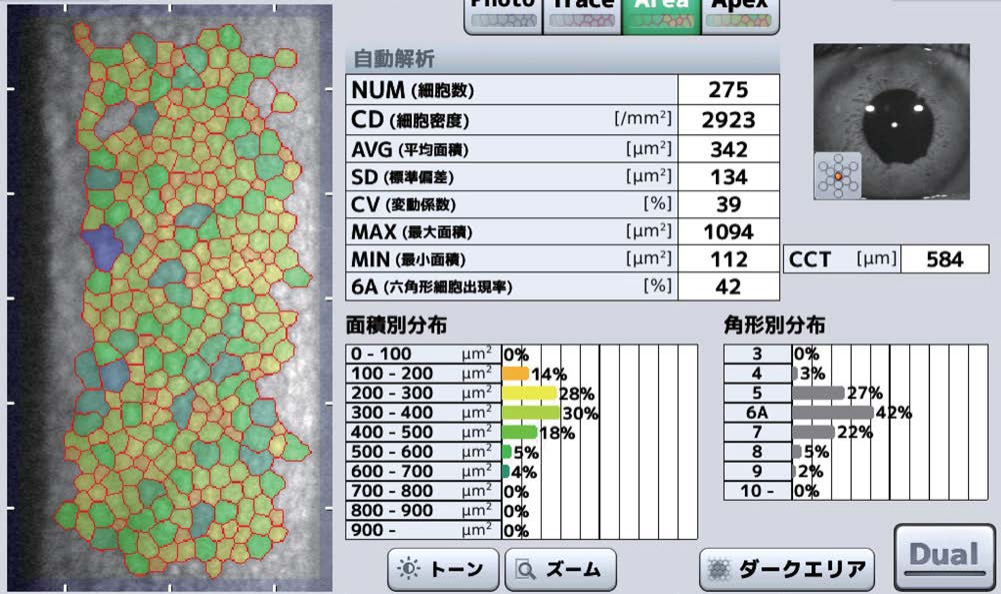
Specular microscopic image taken 1 week after surgery. Corneal endothelial cell density was 2923 cells/mm^2^, showing no significant decrease.

One week after surgery, the best-corrected visual acuity of the operated right eye was 20/16, the intraocular pressure in the right eye was 14 mm Hg, and the corneal endothelial cell density was 2923 cells/mm^2^ (preoperative: 1650 cells/mm^2^; Fig. 2). The central corneal thickness was 580 μm (preoperative: 541 μm), with mild corneal edema.

This study was exempt from approval by the Ethics Committee of the Saitama Red Cross Hospital because it was a retrospective observational study. All the procedures performed in this study adhered to the tenets of the Declaration of Helsinki with written informed consent obtained from the patient.

## 3. Discussion and conclusions

When an IOL that has been dislocated within the capsule is reused and subsequently fixed in the sclera, the removal of the lens capsule surrounding the IOL becomes imperative.^[6]^ Usually, the lens capsule around the IOL is removed using forceps and a vitreous cutter under chandelier lighting, ensuring no damage to the IOL. Additionally, when a lens cortex, such as Soemmering’s ring, is present, its removal can pose challenges. In such cases, a vitreous cutter with a reduced cutting speed rate of 800 cuts per minute^[6]^ or extraction through the corneoscleral wound might be necessary.

In this report, when performing intrascleral fixation of a dislocated intraocular lens by reusing an intracapsular dislocated IOL, the dislocated IOL with the lens capsule was positioned over the iris, and the lens capsule around the IOL was easily removed ab externo by placing the haptics outside the eye through the corneoscleral wound. The advantages of this method are as follows: It is simpler and shorter than the method that uses a vitreous cutter to remove the lens capsule from the vitreous cavity. In some cases, the IOL is found damaged and cannot be reused when the lens capsule around the IOL is removed, thereby exposing it. Even in the case of IOL removal/replacement, the damaged IOL is already placed over the iris, facilitating its removal without the need to repeat previous procedures. Since only a 2.4 mm corneoscleral incision is required, unnecessary incisions are not required, and damage to the intraocular tissues, such as the corneal endothelium and iris, can be minimized.

The technique of effortlessly extracting the capsule and Soemmering’s ring around the IOL through the corneoscleral incision using forceps without damaging the IOL optics is not particularly new. In the era of sutured intrascleral IOL fixation for IOL dislocation, the sutures were traditionally applied ab externo to the haptics.^[3,4]^ In that era, it was a natural concept to place the IOL over the iris once the haptics were placed outside the eye to fix the sutures to the haptics and then the haptics were returned into the eye. However, with the widespread adoption of suture-less intrascleral IOL fixation using a 25-G vitrectomy system, especially in Japan, where vitreous surgeons often perform such surgeries, the lens capsule around the IOL that has fallen into the vitreous cavity is typically removed with a vitrectomy cutter inside the vitreous cavity.

In cases where the lens capsule is partially calcified and hardened, its removal using a vitreous cutter can be both challenging and time-consuming. Furthermore, even with careful use of the vitreous cutter to remove the capsule covering the intraocular lens (IOL) in the vitreous cavity with the aim of preserving the IOL for reuse, damage to the IOL may only become evident upon complete exposure, rendering it unsuitable for reuse.

In contrast, the technique of positioning the IOL haptics outside the eye to facilitate capsule removal offers a more efficient and expeditious solution, even in situations where calcification complicates capsule management with a vitreous cutter. Additionally, if damage to the IOL is discovered only after full exposure, this method remains advantageous, as the IOL optics are already positioned on the iris, allowing for the straightforward removal of the damaged IOL. Given these benefits, the method of placing the haptics outside the eye for lens capsule removal may be recommended.

Nonetheless, this technique is associated with certain limitations. Potential complications include retinal tears or detachments, iris injury, and pupil contraction, particularly if vitreous traction on the lens capsule is inadequately relieved. Although the authors have not observed such severe complications, meticulous care must be taken during surgery to minimize vitreous traction and avoid damage to the iris. In cases of intraocular lens dislocation in a 3-piece IOL, ab externo extraction of the lens capsule is a straightforward and efficient method. If subsequent damage to the IOL is identified, this method allows for easy conversion and replacement. Therefore, in such circumstances, attempting the procedure is a viable option.

## Acknowledgments

We thank Editage (www.editage.com) for English language editing.

## Author contributions

**Conceptualization:** Suguru Nakagawa.

**Data curation:** Suguru Nakagawa.

**Formal analysis:** Suguru Nakagawa.

**Supervision:** Kiyoshi Ishii.

**Writing – original draft:** Suguru Nakagawa.

**Writing – review & editing:** Suguru Nakagawa, Kiyoshi Ishii.

## Supplementary Material


